# Room Temperature Ferromagnetism Engineered in Two-Dimensional Metallic Magnets via Metal–Insulator–Semiconductor Structures

**DOI:** 10.3390/nano16100596

**Published:** 2026-05-13

**Authors:** Yiting Mo, Yijun Huang, Haotian Xu, Shijing Wang, Liang Hu, Lingwei Li

**Affiliations:** Zhejiang Key Laboratory of Energy Conversion Materials for Advanced Motor, Institute of Advanced Magnetic Materials, College of Materials and Environmental Engineering, Hangzhou Dianzi University, Hangzhou 310018, China; 231200006@hdu.edu.cn (Y.M.); 242200066@hdu.edu.cn (Y.H.); 252200084@hdu.edu.cn (H.X.); 252200093@hdu.edu.cn (S.W.)

**Keywords:** MIS structure, 2D magnet, Fe_3_GeTe_2_, optospintronics, charge transfer

## Abstract

The development of novel information-functional devices based on emergent physical phenomena is crucial for integrated circuit technology in the post-Moore era. Two-dimensional magnetic materials present an ideal platform for spintronic devices; however, regulating their room temperature magnetism poses significant challenges. Traditional methods like ionic liquid gating and strain control face issues such as poor stability and complex processes, complicating compatibility with standard silicon technology. Here, we demonstrate a straightforward and robust approach for dielectric layer-engineered room temperature ferromagnetism in 2D metallic magnets by leveraging metal–insulator–semiconductor (MIS) structures. Using surface-oxidized Fe_3_GeTe_2_ as a model system, we systematically investigate how SiO*_x_* dielectric layer thickness (50–300 nm) modulates magnetic properties. Thin dielectric layers significantly enhance room temperature ferromagnetism through boosted interfacial charge transfer, whereas thick layers maintain the material near its intrinsic state due to dielectric screening effects. Furthermore, reversible optical modulation of magnetism is achieved under ultraviolet illumination, with photoresponse capability diminishing as dielectric thickness increases. This work establishes a scalable, silicon-compatible strategy for controlling 2D magnetism and provides critical insights for developing optically tunable spintronic devices and non-volatile memory applications.

## 1. Introduction

In recent years, two-dimensional (2D) magnetic materials have emerged as a novel platform for developing innovative low-dimensional spintronic devices thanks to their atomic-scale long-range magnetic order [1,2,3]. Among these materials, the van der Waals compound Fe_3_GeTe_2_ has attracted considerable attention because of its relatively high bulk Curie temperature (*T*_C_ ≈ 220 K [4]), stable layered structure [5], and adjustable out-of-plane magnetic anisotropy [6,7,8]; its robust ferromagnetic ordering below *T*_C_ stems from the double-exchange interaction between Fe species mediated by Ge and Te atoms. Electrical [4,9,10], mechanical [11,12,13], and optical [14,15,16,17,18,19] methods can effectively enhance and modulate the magnetic properties of Fe_3_GeTe_2_, particularly improving *T*_C_. However, these methods often require continuous external energy supply or complex device structures, in terms of applying external mechanical strain (~4.7%) or high-density charge polarization (~6.9 × 10^14^/cm^2^) to boost perpendicular magnetic anisotropy [9,11]. Alternatively, interface coupling via the proximity effect offers a promising and efficient approach for magnetic regulation [20,21,22,23]. For example, constructing Fe_3_GeTe_2_/FePSe_3_ [16], Fe_3_GeTe_2_/FePS_3_ [24], and Fe_3_GeTe_2_/MnPS_3_ [25] heterojunctions has been shown to significantly enhance the *H*_C_ of Fe_3_GeTe_2_. Nonetheless, these methods have consistently failed to optimize the *T*_C_ of Fe_3_GeTe_2_ to room temperature. At 300 K, intrinsic Fe_3_GeTe_2_ becomes paramagnetic because the temperature exceeds its intrinsic *T*_C_ (~220 K). Rather than inducing ferromagnetism in a non-magnetic material, a more promising route is to enhance and sustain the pre-existing ferromagnetic correlations through interfacial charge engineering [26,27], so that the magnetic signal can persist beyond the bulk *T*_C_.

Integrating 2D magnetic materials with established silicon-based semiconductor processes is a pivotal strategy for tackling computational power and energy efficiency issues in the post-Moore Law era [4,28,29,30,31,32]. In this context, designing and enhancing the magnetic properties of 2D magnetic materials on silicon substrates is vital for advancing silicon-based 2D spintronic devices [4,33,34,35,36]. Previous research has shown that leveraging work function differences between materials to create built-in electric fields at heterointerfaces can effectively modulate carrier concentrations [16,18,24,37,38,39,40,41] and enable efficient electric field modulation of magnetism [24,33,42]. Further studies suggest that illumination can also influence charge transfer directions [14,18,43,44], providing insights for developing light-controlled magnetic devices. However, in the traditional Si/SiO*_x_* substrate system, the SiO*_x_* layer has typically been viewed as a passivating dielectric layer [45]. The coupling mechanism between Si/SiO*_x_* and 2D magnetic materials and its effect on magnetic properties is not well understood. It remains unclear how factors such as SiO*_x_* thickness and interfacial state distribution affect magnetic exchange interactions among itinerant electrons in Fe_3_GeTe_2_, which impedes effective control of magnetic properties in this system.

The SiO*_x_* layer contains significant fixed parasitic charges, which notably affect the spin–charge distribution in Fe_3_GeTe_2_ [46,47,48,49], especially when the SiO*_x_* layer is very thin. These surface-pinned states and parasitic charges have a considerable impact on the carrier distribution and spin ordering in the overlying 2D metallic magnet [26,27,34,50]. The study here proposes a method to effectively regulate the room temperature magnetism of surface-oxidized Fe_3_GeTe_2_ by controlling the thickness of the SiO*_x_* dielectric layer. When the critical thickness of the dielectric layer is below 300 nm, a pronounced room temperature magnetic force microscope (MFM) signal is observed in moderately thin Fe_3_GeTe_2_ (15–20 nm) nanosheets obtained through mechanical exfoliation and transfer procedure. This ferromagnetic signal is strongly correlated with the SiO*_x_* layer thickness, with thinner layers showing enhanced magnetic properties. Through ultraviolet (UV) irradiation experiments, we confirm that this magnetization enhancement is closely linked to interfacial charge transfer, a process described by the proximity polarization effect in the metal–insulator–semiconductor (MIS) structure [8,24,25,51,52,53]. This discovery not only uncovers a novel 2D magnetism regulation mechanism compatible with silicon-based industry but also provides a crucial foundation for developing Fe_3_GeTe_2_-based spintronic devices capable of operating at room temperature.

## 2. Materials and Methods

### 2.1. Sample Preparation

Fe_3_GeTe_2_ single crystal (99.99%, SixCarbon Technology, Shenzhen, China) was mechanically exfoliated using a commercial blue membrane (Nitto, SPV-224R, Osaka, Japan) in an Ar-filled glove box with water and oxygen concentration below 0.1 ppm. Utilizing van der Waals forces between polydimethylsiloxane (PDMS, Zhongke Experimental Materials, Hefei, China), a few nanosheets adhered to the blue membrane were transferred onto the PDMS stamp. This stamp was then applied to Si/SiO*_x_* wafers with different oxide layer thicknesses and heated to 60 °C for 10 min by a micromanipulator (METATEST, E1-G, Nanjing, China). This heating process weakened the van der Waals forces between the stamp and the nanosheets, facilitating their transfer onto the Si/SiO*_x_* surfaces. Given that the SiO*_x_* layer thickness is closely linked to its charge polarization and UV light (*λ* = 365 nm) modulation capacities for Fe_3_GeTe_2_ magnetism, we selected dielectric layers of varying thicknesses (50–300 nm) to examine thickness-dependent effects. The specific SiO*_x_* thickness values in single-sided polished Si/SiO*_x_* wafers (PrMat Technology, Shanghai, China) were 50 nm, 100 nm, 200 nm, and 300 nm, respectively, with *p*-Si(100) resistivity below 0.01 Ω cm.

### 2.2. Characterization

The quality of Fe_3_GeTe_2_ single crystal was characterized using an X-ray diffractometer (XRD, Rigaku, SmartLab, Tokyo, Japan). Fundamental magnetic properties were measured using a superconducting quantum interference device (SQUID, Quantum Design, MPMS-7T, San Diego, CA, USA) under zero-field-cooling (ZFC)/field-cooling (FC) modes. The hysteresis loops at 300 K for Fe_3_GeTe_2_ nanosheets dispersed on Si/SiO*_x_* substrates were quantified using a polar magneto-optical Kerr microscope (MOKE, Truth Instruments, KMP-L, Qingdao, China). To investigate the interfacial quality between Fe_3_GeTe_2_ and SiO*_x_* layers, cross-sectional high-angle annular dark-field scanning transmission electron microscopy (HAADF-STEM, FEI, Talos F200S, Hillsboro, OR, USA) and energy-dispersive X-ray spectroscopy (EDS) line-scan analysis were performed.

Atomic force microscopy (AFM, JPK Instruments, NanoWizard 4-NanoScience, Berlin, Germany) was employed to analyze the thickness of exfoliated Fe_3_GeTe_2_ samples. And its Kelvin probe force (KPFM) and magnetic force (MFM) modes are utilized to determine the contact potential difference (CPD) and shifted lock-in phase angles between the sample surface and the Pt/Ir- (NanoSensors, PPP-EFM, Neuchatel, Switzerland) and Co/Cr-coated probe (NanoSensors, PPP-LC-MFMR), respectively. Raman spectra were recorded in a confocal microscopic Raman spectrometer (METATEST, MStarter 100, Najing, China) with an excitation wavelength of 532 nm. The MFM lock-in phase and KPFM surface potential values were extracted using a standardized protocol to ensure reproducibility. For each nanosheet, a rectangular region of interest (ROI) of ~2 × 2 μm^2^ was defined in the central area, avoiding edges and topographic defects. The substrate background was determined by averaging signals from adjacent bare SiO*_x_* regions (~3 μm from the nanosheet edge) and subtracted from the nanosheet ROI. Pixel values within the ROI were averaged after applying a Gaussian smoothing filter (3 × 3 kernel) to suppress high-frequency noise. Dark and illumination measurements were performed on identical ROIs, with tip position verified by AFM topography. Error bars originate from the standard deviation from 5–6 spatial measurements across the nanosheet.

## 3. Results and Discussion

**Figure 1:**
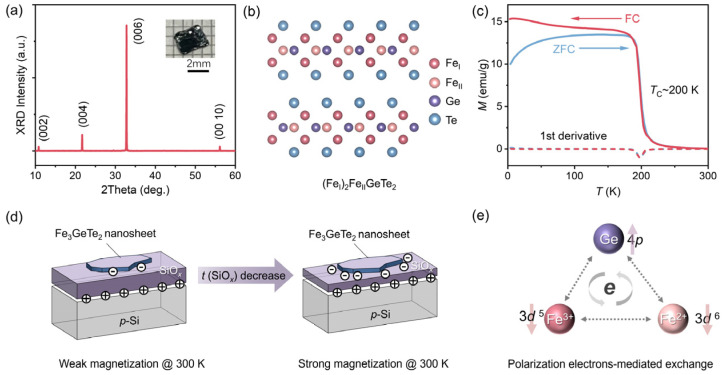
Structure, magnetic property, and proximity-polarized magnetization enhancement of Fe_3_GeTe_2_. (**a**) Indexed XRD pattern of Fe_3_GeTe_2_ single crystal. Inset is the digital photograph of a bulk crystal with a lateral size of around 3 mm. (**b**) Layered crystal structure of Fe_3_GeTe_2_ containing two inequivalent Fe_I_ and Fe_II_ species. (**c**) Temperature-dependent magnetization curves of Fe_3_GeTe_2_ single crystal under ZFC and FC conditions and their first derivative plots for determining *T*_C_. The applied field during FC measurement is set as 1000 Oe. (**d**) Schematic demonstration of the electron polarization phenomenon of an individual Fe_3_GeTe_2_ nanosheet on the Si/SiO*_x_* substrate with decreasing dielectric layer thickness. (**e**) Indirect double exchange process mediated by polarization electron hopping among Fe_I_-Ge-Fe_II_.

As depicted in Figure 1a, the XRD pattern reveals sharp (00*L*) crystal plane peaks, confirming that the material has a hexagonal crystal structure with the *P*6_3_/mmc space group and good crystalline quality [26], with no impurity phases other than Fe_3_GeTe_2_ observed. The crystal structure schematic diagram in Figure 1b further shows that the structure consists of covalently bonded Fe-Ge layers sandwiched between two Te atomic layers, with a van der Waals interlayer gap of approximately 2.95 Å [4]. There are two inequivalent Fe sites (Fe_I_ and Fe_II_, referring to Fe^3+^ and Fe^2+^ species, respectively), together contributing to a superexchange process with the aid of Ge and Te atoms. Due to the weak interlayer binding force, thinning the bulk along the c-axis is relatively easy, a structural feature that enables the acquisition of nanosheets via mechanical exfoliation. Fe_3_GeTe_2_ is an itinerant ferromagnetic metal, where each layer exhibits ferromagnetic spin alignment and significant perpendicular anisotropy. The spin alignment between layers can be either parallel (ferromagnetic) or antiparallel (antiferromagnetic) [54]. Generally, the ferromagnetic superexchange interaction via Fe-Te-Fe and Fe-Ge-Fe is stronger than the antiferromagnetic direct exchange between Fe-Fe, resulting in overall ferromagnetic order in bulk Fe_3_GeTe_2_ below *T*_C_. To verify the intrinsic ferromagnetism of Fe_3_GeTe_2_, we measured its fundamental magnetic properties. As shown in Figure 1c, the abrupt change in the ZFC and FC curves and the characteristic peak in the first derivative together determine *T*_C_ ≈ 200 K, indicating the magnetic phase transition from ferromagnetism to paramagnetism. This magnetic transition temperature coincides with most reported values and is closely dependent on the stoichiometric ratio of Fe atoms [9]. Since the magnetism of Fe_3_GeTe_2_ can be regulated by electrons [41], we chose heavily doped *p*-type Si as the semiconductor layer and the SiO*_x_* layer as the insulator layer to construct a classic metal–insulator–semiconductor (MIS) regulation structure. When the SiO*_x_* layer is thin enough, a high density of adsorbed states is present in the surface region of SiO*_x_* for filling positively charged traps. In this regard, polarized electrons are accumulated on the SiO*_x_* side in the MIS structure. When Fe_3_GeTe_2_ is placed on the SiO*_x_* surface, the nanosheets acquire these induced surface electrons and become spin-polarized. Figure 1d shows that placing Fe_3_GeTe_2_ nanosheets with proper thickness (15–20 nm) onto Si/SiO*_x_* substrates with varying dielectric layer thicknesses demonstrates that modulating the SiO*_x_* layer thickness alters charge doping efficiency. This, in turn, regulates the neighboring polarization intensity applied to Fe_3_GeTe_2_, enabling effective and non-volatile control of its room temperature ferromagnetism. This approach provides a viable pathway for silicon-based integration of 2D magnetic materials.

Before presenting the thickness-dependent results, we clarify the rationale for selecting Fe_3_GeTe_2_ nanosheets with a thickness of 15–20 nm. This range represents an optimal compromise for observing the interfacial charge polarization effect: nanosheets thinner than ~15 nm are challenging to transfer intact and tend to exhibit structural degradation during the thermal transfer process, whereas thicker nanosheets (>20 nm) reduce the surface-to-volume ratio and weaken the interfacial charge transfer efficiency. We note that the induced charges primarily reside in the outermost layers near the Fe_3_GeTe_2_/SiO*_x_* interface; consequently, thinner nanosheets would exhibit stronger modulation due to higher charge density per unit volume, but their mechanical instability limits practical use. The 15–20 nm range ensures both sufficient signal contrast for reliable detection and structural integrity of the samples. Figure 1e illustrates the key ferromagnetic exchange mechanism in this material: the spin-majority electrons in Fe^3+^ and Fe^2+^ atoms undergo indirect exchange with the spin-minority electrons of the Ge atom, promoting majority spins to hop in an antiparallel configuration to the Ge 4*p* orbital, and subsequently, driven by thermal fluctuations, occupy the empty 3*d* orbitals of Fe^2+^, thereby establishing a robust ferromagnetic state.

**Figure 2:**
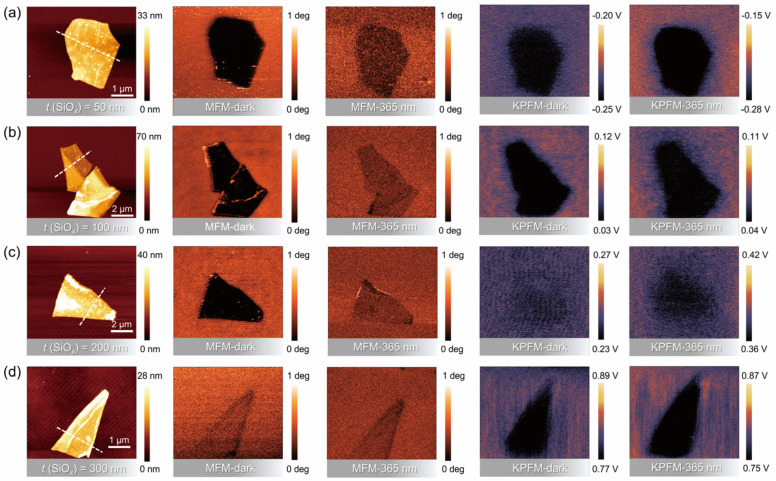
AFM morphology, MFM, and KPFM images of Fe_3_GeTe_2_ nanosheets on Si/SiO*_x_* substrates under dark and 365 nm UV illumination conditions. The SiO*_x_* layer thicknesses are (**a**) 50 nm, (**b**) 100 nm, (**c**) 200 nm, and (**d**) 300 nm, respectively.

**Figure 3:**
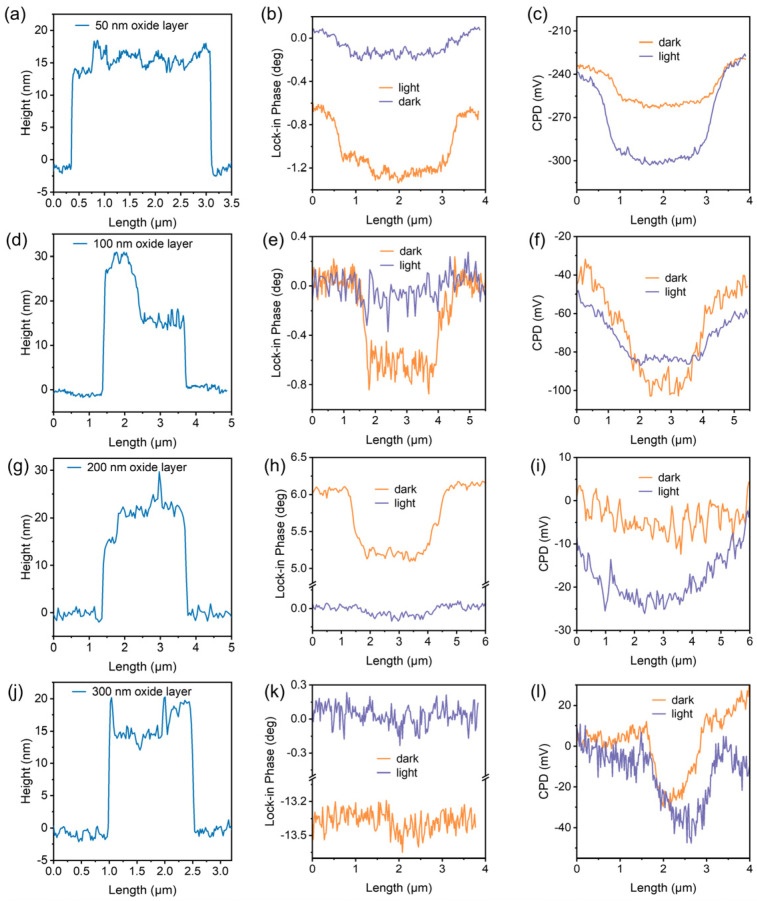
Comparison of AFM thickness, MFM lock-in phase, and KPFM surface potential differences under dark and 365 nm UV illumination conditions. AFM height (**a**,**d**,**g**,**j**), MFM lock-in phase (**b**,**e**,**h**,**k**), and KPFM surface potential (**c**,**f**,**i**,**l**) images of samples with SiO*_x_* layer thicknesses of 50 nm (**a**–**c**), 100 nm (**d**–**f**), 200 nm (**g**–**i**), and 300 nm (**j**–**l**). All the profile data are collected from the corresponding white dashed lines shown in Figure 2. The *y*-axis scales in panels (**b**,**c**,**e**,**f**,**h**,**i**,**k**,**l**) have been individually optimized to clearly visualize the illumination-induced changes, with explicit numerical annotations provided for dark and illumination values.

Given the sensitivity of Fe_3_GeTe_2_ magnetism to its thickness, we chose nanosheets with a thickness of about 15–20 nm (for details, see later) for the next study because such a thickness easily induces a considerable proximity effect. Under dark conditions (see “MFM-dark” panel in Figure 2a), we observed marked darkening of MFM lock-in phase contrast in Fe_3_GeTe_2_ nanosheets, indicating a magnetically attractive force between the sample surface and probe tip. There is a maximum phase contrast difference of approximately 0.71° compared to the diamagnetic Si/SiO*_x_* substrate, which corresponds to the case of a 50 nm dielectric layer thickness. It is noteworthy that the MFM measurements were implemented at room temperature (300 K), which suggests an enhanced magnetization effect, as Fe_3_GeTe_2_ typically only exhibits ferromagnetism at low temperatures. This emerging weak ferromagnetism might stem from the strain coupling or the charge doping effect. However, the occurrence of room temperature ferromagnetism in Fe_3_GeTe_2_ by means of strain application requires at least a 2% strain level, which is impossible for flat silicon substrates through mild thermal transfer. Considering the presence of a high density of charge pinning states on the surface of the SiO*_x_* layer, unintentional doping of electrons from the Si/SiO*_x_* substrate will become a key factor that cannot be overlooked. We further exposed the sample to 365 nm UV light, as shown in the “MFM-365 nm” panel of Figure 2. The black region in the MFM image noticeably diminished, and the phase contrast difference decreased to about 0.19° (see Figure 3b). This change is reversible when the UV illumination is switched off, indicating that the origin of magnetism might be closely correlated with a light-sensitive charge transfer process.

To further elucidate this charge transfer behavior of Fe_3_GeTe_2_ on 50 nm SiO_x_-coated silicon wafers, we characterized the surface potential under dark and light conditions using KPFM, as shown in the “KPFM-dark” and “KPFM-365 nm” panels in Figure 2a. The contact potential difference (CPD) between the AFM tip and the sample surface can be expressed as *V*_CPD_ = (*Φ*_tip_ − *Φ*_sample_)/*e*, where *Φ*_tip_ and *Φ*_sample_ are the work functions of the tip and sample, respectively. Hence, the work function difference between the Fe_3_GeTe_2_ nanosheet and the SiO*_x_* surface can be expressed as Δ*Φ* = *Φ*_nanosheet_ − *Φ*_SiO_*_x_* = *e* [*V*_CPD-nanosheet_ − *V*_CPD-SiO_*_x_*] = *e*Δ*V*_CPD_. Considering *V*_CPD-nanosheet_ < *V*_CPD-SiO_*_x_*, *Φ*_nanosheet_ becomes smaller than *Φ*_SiO_*_x_*. Under dark conditions, *V*_CPD_ is approximately −30 mV (averaged from the central ROI with background subtraction; see Methods), which is a very small difference (0.03 eV) in Fermi level (*E*_F_), indicating that the SiO*_x_* surface possesses abundant fixed positively charged traps that can gain electrons from adsorbed molecules or groups and elevate surface *E*_F_. According to the plane-parallel capacitor principle, some of the polarization-induced electrons are involved/doped into nanosheets and eventually result in a weak ferromagnetic state. Under UV illumination, Δ*V*_CPD_ shifts to approximately –60 mV. The absolute difference in Δ*V*_CPD_ before and after illumination is about 30 mV, suggesting that illumination can drive a significant charge transfer between Fe_3_GeTe_2_ and Si/SiO*_x_* and induce the room temperature optical demagnetization in MFM.

As the dielectric layer thickness increases, for instance, for the Fe_3_GeTe_2_ nanosheet on the Si/SiO*_x_* substrate with a 100 nm oxide layer, the MFM contrast in dark differed from the substrate by approximately 0.66° (see Figure 3e), which is slightly lower than that observed for the sample on the substrate with a 50 nm oxide layer. This reveals that the polarization capability is accordingly reduced. Upon UV irradiation, the magnetic contrast further weakened to approximately 0.05° (see Figure 3e), leaving the sample in a slightly magnetic state. The corresponding KPFM results in Figure 3f demonstrate that the absolute change in Δ*V*_CPD_ before and after illumination is approximately 23 mV, which is also smaller than the case of 50 nm thickness. As for the Fe_3_GeTe_2_ nanosheet attached to the SiO*_x_* layer with a thickness of 200 nm, the phase contrast difference in dark-field MFM is about 0.52° and further decreases to 0.04° after UV irradiation (see Figure 3h). The absolute value of Δ*V*_CPD_ measured by KPFM is also reduced to 10 mV, as shown in Figure 3i. This demonstrates a consistent charge transfer-type optical demagnetization mechanism, with the polarization capability gradually decreasing with increasing dielectric layer thickness. Ultimately, we examined the case of 300 nm oxide-coated silicon wafers (the most commonly observed specification in the commercial market). Figure 3j displays a similar Fe_3_GeTe_2_ thickness of approximately 16 nm. Under dark-field conditions (Figure 3k), its magnetic phase contrast is only about 0.06°, which is near the noise magnetic signal level and significantly lower than that in other dielectric layer thicknesses. It can be expected that, when the dielectric layer exceeds a certain critical thickness, the number of induced electrons significantly decreases and the proximity polarization effect becomes weak due to the dielectric screening effect. In this regard, despite light irradiation, the contrast change is minimal. The KPFM in Figure 3l also reveals extremely similar potential differences under dark and light fields, indicating that there is almost no charge transfer between Fe_3_GeTe_2_ and the Si/SiO*_x_* substrate, maintaining a near-intrinsic state. The above observations collectively demonstrate that charge transfer decreases significantly with increasing dielectric layer thickness. A thick dielectric layer effectively shields against electric fields from fixed charges and weakens the proximity polarization effect. Therefore, for applications aiming to preserve the intrinsic magnetism of Fe_3_GeTe_2_, thicker dielectric layers or other inert substrates (e.g., *h*-BN) are recommended. Conversely, for devices pursuing electrically/optically controlled magnetic modulation, thin dielectric layers should be employed to construct MIS structures, enabling efficient charge doping and room temperature magnetic control.

**Figure 4:**
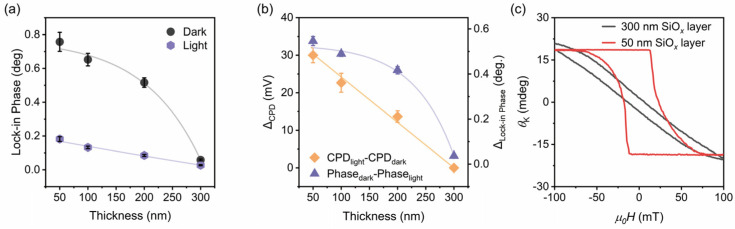
Scatter plots of differences against dielectric layer thickness under dark and 365 nm UV illumination conditions. (**a**) MFM lock-in phase; (**b**) KPFM surface potential. Error bars represent the standard deviation from multiple spatial measurements across the nanosheet. Solid curves are guides for the eye. (**c**) Polar MOKE hysteresis loops measured at 300 K for Fe_3_GeTe_2_ nanosheets on Si/SiO*_x_* substrates with 50 nm (red) and 300 nm (black) oxide thicknesses.

Figure 4 summarizes the thickness-dependent evolution of the MFM lock-in phase and KPFM surface potential in Fe_3_GeTe_2_ nanosheets. As shown in Figure 4a, both the dark-state phase (black circles) and the illumination-state phase (purple hexagons) exhibit monotonic decreases with increasing SiO*_x_* thickness, with error bars confirming the reproducibility of spatial measurements. Notably, the dark-state phase decreases from ~0.75° at 50 nm to ~0.06° at 300 nm, approaching the instrumental noise floor (~0.02°), while the illumination-state phase similarly decreases from ~0.18° to ~0.02°. This consistent thickness dependence strongly correlates with the dielectric screening-modulated magnetic proximity effect.

Figure 4b further reveals that both the CPD difference (CPD_light_ − CPD_dark_, orange diamonds) and the lock-in phase difference (Phase_dark_ − Phase_light_, purple triangles) decrease monotonically with dielectric thickness. The CPD difference decreases from ~30 mV at 50 nm to near zero at 300 nm, while the phase difference decreases from ~0.55° to ~0.04°. The close correlation between these two independent measurements, both showing similar decay trends, further validates the pivotal role of interfacial charge transfer in the light-controlled magnetization process. The non-zero error bars at each thickness point reflect the spatial inhomogeneity of interfacial charge distribution, which is more pronounced at thinner dielectric layers due to stronger field penetration. The fixed charge at the interface may act as a built-in gate voltage and then estimate the surface density of charge (Δ*n*) transferred to the Fe_3_GeTe_2_ using the MIS capacitor formula. Assuming fixed charge *Q*_f_ = 3 × 10^12^ cm^−2^ (typical value for thermal oxidation silicon) and dielectric layer thickness *d* = 50 nm, |*V*_FB_| ≈ *Q*_f_ *d*/*ε*_ox_*ε*_0_ ≈ 4.4 V, where *ε*_ox_ and *ε*_0_ denote the permittivity of SiO*_x_* and vacuum, respectively. Thereby, the induced Δ*n* can be estimated as Δ*n* = *C*_ox_ |*V*_FB_|/*e* ≈ 1.9 × 10^13^ cm^−2^, very close to the critical threshold for activating room temperature ferromagnetism in Fe_3_GeTe_2_ [9]. These statistical findings quantitatively reveal how dielectric layer thickness continuously modulates the room temperature magnetism and optical demagnetization behavior of Fe_3_GeTe_2_. The observation here provides clear guidance for optimizing the dielectric layer design in silicon-based spintronic devices.

The error bars in Figure 4 warrant further discussion. The relatively larger error bars at 50 nm and 100 nm dielectric thicknesses (particularly for CPD measurements) reflect the stronger spatial variation of interfacial charge trapping at thin dielectrics, where the electric field penetration is more sensitive to local defect distribution. In contrast, the smaller error bars at 200 nm and 300 nm indicate more uniform charge distribution due to effective dielectric screening. This thickness-dependent error bar behavior is consistent with the physical picture of MIS-mediated charge transfer and further supports the reliability of our conclusions.

To directly correlate the interfacial charge transfer inferred from KPFM with macroscopic magnetic order, we performed polar MOKE measurements on Fe_3_GeTe_2_ nanosheets on Si/SiO*_x_* substrates with 50 nm and 300 nm oxide thicknesses. As shown in Figure 4c, the 50 nm sample exhibits a distinct asymmetric hysteresis loop with a large coercive field (*H*_C_ ≈ 200 Oe) and a pronounced loop shift, while the 300 nm sample shows a nearly symmetric loop with a much smaller *H*_C_ ≈ 90 Oe. The ~5 nm interfacial oxidation layer (denoted as O-Fe_3_GeTe_2_), formed through natural surface oxidation upon exposure to the ambient environment, exhibits amorphous antiferromagnetic insulating characteristics [55,56,57]. Naturally oxidized Fe_3_GeTe_2_ shows clear antiferromagnetic ordering, producing significant exchange bias effects at the FM/AFM interface [55]. The O-Fe_3_GeTe_2_ phase is an antiferromagnetic insulator because oxygen incorporation induces negative spin polarization via spin–orbit coupling, mediating antiferromagnetic interlayer coupling [57,58]. In the Fe_3_GeTe_2_ nanosheet on the Si/SiO*_x_* substrate with a 50 nm oxide layer, the strong built-in electric field from the thin SiO*_x_* layer penetrates through the O-Fe_3_GeTe_2_ interlayer, enhancing the interfacial magnetic anisotropy [8]. This enhanced anisotropy stabilizes the exchange bias effect at the ferromagnetic Fe_3_GeTe_2_/antiferromagnetic O-Fe_3_GeTe_2_ interface, which in turn produces the asymmetric hysteresis loop and enhanced coercivity [55,56,57]. In contrast, in the Fe_3_GeTe_2_ nanosheet on the Si/SiO*_x_* substrate with a 300 nm oxide layer, dielectric screening suppresses this field penetration; although the O-Fe_3_GeTe_2_ layer remains structurally similar, the built-in electric field cannot effectively polarize, resulting in a near-intrinsic weak ferromagnetic state. For Fe_3_GeTe_2_ with an ultrathin surface dielectric layer (typical thickness ~5 nm), the observed SiO*_x_* thickness-dependent magnetic responses indicate that the thickness of the composite dielectric layer composed of SiO*_x_* and O-Fe_3_GeTe_2_ governs the charge transfer efficiency across the interface, which in turn determines the magnetic behavior. The O-Fe_3_GeTe_2_ layer functions as an antiferromagnetic charge mediation bridge whose polarization efficiency is controlled by the underlying SiO*_x_* thickness, rather than acting as an independent magnetic source.

**Figure 5:**
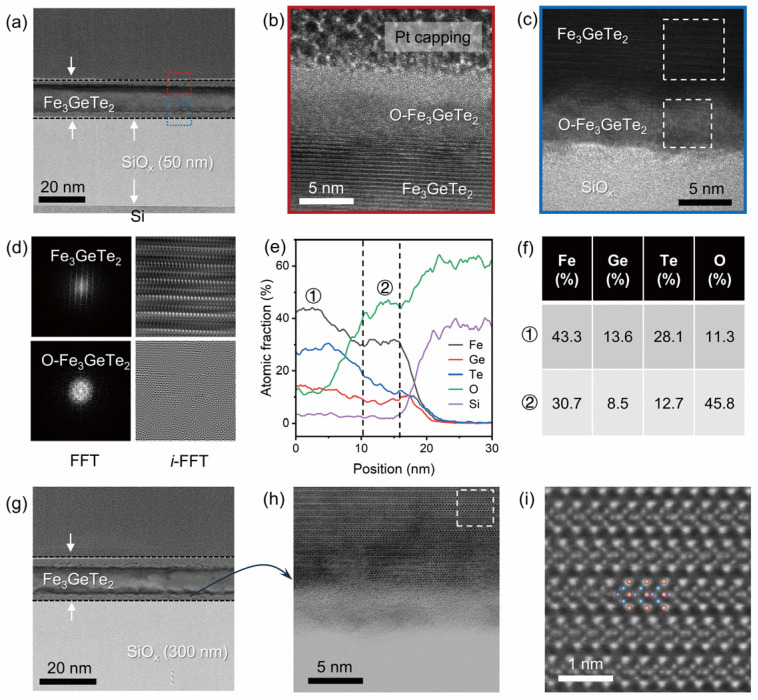
Cross-sectional HAADF-STEM characterization and interfacial structure comparison of surface-oxidized Fe_3_GeTe_2_ nanosheets on Si/SiO*_x_* substrates with 50 nm and 300 nm oxide thicknesses. (**a**) Low-magnification cross-sectional TEM overview image of the surface-oxidized Fe_3_GeTe_2_ nanosheets on Si/SiO*_x_* substrates with a 50 nm oxide layer. White arrows mark the interface positions, and red and blue boxes indicate the magnified regions in (**b**,**c**), respectively. (**b**) High-resolution TEM image of the red-boxed region in (**a**), showing from top to bottom the Pt capping layer, ~5 nm amorphous O-Fe_3_GeTe_2_ oxidation layer, and crystalline Fe_3_GeTe_2_. (**c**) High-resolution TEM image of the blue-boxed region in (**a**), with white dashed boxes marking the areas for Fast Fourier Transform (FFT) analysis. (**d**) Upper row: FFT diffraction pattern (exhibits periodic diffraction spots) and inverse FFT (*i*-FFT) image (clear atomic lattice) of the crystalline Fe_3_GeTe_2_ region. Lower row: FFT (diffuse ring pattern, confirming amorphous structure) and *i*-FFT image of the O-Fe_3_GeTe_2_ oxidation layer. (**e**) EDS elemental line-scan profiles, where region ① corresponds to the crystalline Fe_3_GeTe_2_ region and region ② corresponds to the O-Fe_3_GeTe_2_ interfacial oxidation layer. (**f**) Quantitative EDS atomic percentages for regions ① and ②, showing significantly increased oxygen content in the oxidation layer while the stoichiometric ratio of Fe, Ge, and Te is largely maintained. (**g**) Low-magnification cross-sectional TEM overview image of the Fe_3_GeTe_2_ nanosheet on the Si/SiO*_x_* substrate with a 300 nm oxide layer. (**h**) Aberration-corrected HAADF-STEM-magnified image, enlarged from the region indicated by the arrow in (**g**), with the white dashed box marking the magnified region in (**i**). (**i**) Atomic-resolution TEM image showing the atomic arrangement in the Fe_3_GeTe_2_ crystalline region, with colored overlays representing the fitted atomic framework model to demonstrate the match between the crystal structure and experimental image.

To gain insight into the interfacial structure between Fe_3_GeTe_2_ and the SiO*_x_* layer, we performed cross-sectional HAADF-STEM characterization on Fe_3_GeTe_2_ nanosheets on Si/SiO*_x_* substrates with 50 nm and 300 nm oxide layers. As shown in Figure 5a, the low-magnification TEM overview image of the nanosheet on the substrate with a 50 nm SiO*_x_* layer clearly reveals a sandwich structure composed of a Pt capping layer, the surface O-Fe_3_GeTe_2_ layer, and the Fe_3_GeTe_2_ layer. Figure 5b, a magnified TEM image of the red-boxed region in Figure 5a, further reveals a ~5 nm amorphous O-Fe_3_GeTe_2_ oxidation layer between the Pt protective capping layer and the crystalline Fe_3_GeTe_2_. This oxidation layer exhibits diffuse spot contrast, in sharp contrast to the clear lattice fringes of the underlying Fe_3_GeTe_2_. Figure 5c, a magnified TEM image of the blue-boxed region in Figure 5a, shows a similar ~5 nm O-Fe_3_GeTe_2_ oxidation layer between Fe_3_GeTe_2_ and SiO*_x_*, further confirming the existence and spatial continuity of this interfacial oxidation layer. To verify the structural characteristics of the oxidation layer, we performed FFT analysis on both the crystalline Fe_3_GeTe_2_ region and the O-Fe_3_GeTe_2_ oxidation layer (Figure 5d). The first row shows that the crystalline Fe_3_GeTe_2_ region exhibits periodic crystalline diffraction spots, and the *i*-FFT image displays a clear layered atomic arrangement. The second row shows that the FFT of the O-Fe_3_GeTe_2_ oxidation layer presents a diffuse ring pattern, and the *i*-FFT image shows no discernible atomic number contrast, confirming the amorphous nature of this oxidation layer. EDS elemental line scanning (Figure 5e) quantitatively reveals the elemental distribution across the interface. Region ① corresponds to the crystalline Fe_3_GeTe_2_ region, where Fe, Ge, and Te elements maintain relatively stable atomic fractions. Region ② corresponds to the O-Fe_3_GeTe_2_ interfacial oxidation layer, where the O content rises sharply to a peak while Fe, Ge, and Te gradually decrease but remain coexistent. The gradual elemental transition indicates partial surface oxidation rather than complete phase decomposition. Quantitative EDS analysis (Figure 5f) further shows that the atomic composition of region ① is Fe 43.3%, Ge 13.6%, Te 28.1%, and O 11.3%, close to the stoichiometric ratio of Fe_3_GeTe_2_; region ② has a composition of Fe 30.7%, Ge 8.5%, Te 12.7%, and O 45.8%, where oxygen is dominant but the stoichiometric ratio of Fe, Ge, and Te is largely maintained.

To verify the universality of the interfacial structure, we performed the same cross-sectional characterization on the Fe_3_GeTe_2_ nanosheet on the Si/SiO*_x_* substrate with a 300 nm oxide layer. As shown in Figure 5g, the nanosheet on the substrate with a 300 nm SiO*_x_* layer clearly reveals a sandwich structure, with a distinct O-Fe_3_GeTe_2_ interfacial layer formed between the Fe_3_GeTe_2_ layer and the SiO*_x_* layer. The aberration-corrected HAADF-STEM-magnified image (Figure 5h) reveals similar amorphous characteristics at the interface region. The atomic-resolution TEM image (Figure 5i) displays the clear atomic arrangement in the Fe_3_GeTe_2_ crystalline region, with colored overlays of the fitted atomic framework model matching well with the experimental image, confirming the structural integrity of surface-oxidized Fe_3_GeTe_2_ on the 300 nm SiO*_x_* layer.

**Figure 6:**
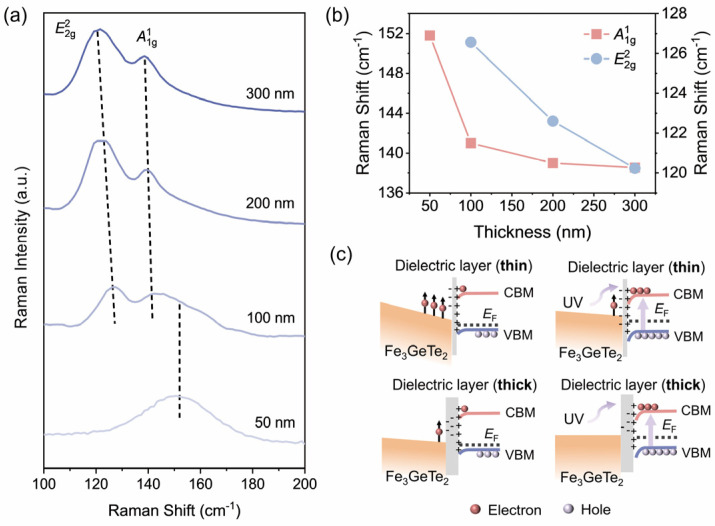
Phonon vibration analysis and proximity polarization mechanism of Fe_3_GeTe_2_ on Si/SiO_x_ substrates with different dielectric layer thicknesses. (**a**) Raman spectral evolution of Fe_3_GeTe_2_ nanosheets, showing the complete disappearance of the in-plane $E_{2g}^{2}$ mode (~125.3 cm^−1^) at 50 nm due to strong interfacial charge transfer, while the out-of-plane $A_{1g}^{1}$ mode (~142.6 cm^−1^) persists with a large red shift. (**b**) Dielectric layer thickness-dependent peak position changes for the $E_{2g}^{2}$ and $A_{1g}^{1}$ modes. (**c**) Schematic of charge trap-driven surface energy band bending and magnetization enhancement in Fe_3_GeTe_2_. The distinct mode responses reflect the different sensitivities of in-plane (Fe-Ge) and out-of-plane (Te-Te) vibrations to interfacial charge doping.

Figure 6 further reveals the interfacial regulation mechanism of different SiO*_x_* thicknesses on Fe_3_GeTe_2_ from the perspectives of lattice vibrations and electronic structure. As depicted in Figure 6a, the systematic changes in the Raman spectra clearly reflect the influence of interfacial coupling on lattice vibrations. As the dielectric layer thickness increases from 50 nm to 300 nm, the intensity ratio between the $A_{1g}^{1}$ peak (representing out-of-plane vibration of Te-Te bonds, around 142.6 cm^−1^ [54]) and the $E_{2g}^{2}$ peak (representing in-plane vibration of Fe-Ge bonds, around 125.3 cm^−1^ [9]) changes significantly. Particularly noteworthy is the complete disappearance of the $E_{2g}^{2}$ peak in the 50 nm thin dielectric layer, while the $A_{1g}^{1}$ peak shows a significant red shift, Δ*ω* = 13.27 cm^−1^. This phenomenon indicates that interfacial charge transfer can effectively modulate lattice vibration through electron–phonon coupling [59]. Further analysis of the Raman peak position, as clearly shown in Figure 6b, reveals that with increasing dielectric layer thickness, both peaks gradually have a red shift. This thickness-dependent phonon softening behavior is highly consistent with the aforementioned interfacial coupling theory, indicating weakened charge transfer with increasing dielectric layer thickness. The band structure in Figure 6c provides a possible explanation for this regulation mechanism. For the thin dielectric layers, fixed charge traps can generate a built-in electric field towards the Fe_3_GeTe_2_ side and induce the band bending upward from the interface to the bulk region. The generated electronic pocket accumulates enough electrons to polarize high-temperature ferromagnetic exchange interaction in Fe_3_GeTe_2_. Optically excited carriers can screen trap states and eventually weaken interfacial charge transfer between Fe_3_GeTe_2_ and the dielectric layer, which provides a theoretical explanation for the observed photo-induced demagnetization phenomenon. In contrast, a thick dielectric layer effectively shields against electric fields from fixed charges and weakens the proximity polarization effect, enabling the band structure close to the intrinsic state. To sum up, the above discussion collectively demonstrates that the dielectric layer thickness not only regulates the magnetism of Fe_3_GeTe_2_ through interfacial charge transfer but also affects its lattice dynamics through electron–phonon coupling. In addition, the systematic red shift of the Raman peaks also reflects the lattice strain induced by interfacial interactions, while the synergy of the charge and strain may offer a complete microscopic physical picture for understanding these effects. This discovery establishes an internal link between phonon behavior, electronic structure, and magnetic properties, providing a multi-physics coupling research perspective for regulating 2D magnetic materials through dielectric environment design.

The distinct responses of the $E_{2g}^{2}$ and $A_{1g}^{1}$ modes to interfacial charge transfer warrant further mechanistic clarification. The $E_{2g}^{2}$ mode (~125.3 cm^−1^) corresponds to in-plane vibration of Fe-Ge bonds and is highly sensitive to in-plane lattice strain and interfacial charge doping [54,59]. In the Fe_3_GeTe_2_ nanosheet on the Si/SiO*_x_* substrate with a 50 nm SiO*_x_* layer, strong electron–phonon coupling induced by interfacial charge transfer substantially softens this mode, and the accumulated electrons in Fe 3*d* orbitals weaken the Fe-Ge in-plane bonding sufficiently to suppress the $E_{2g}^{2}$ peak below the detection limit. This is consistent with theoretical predictions that heavy charge doping can drive structural instabilities in 2D ferromagnets [9,41]. In contrast, the $A_{1g}^{1}$ mode (~142.6 cm^−1^) represents out-of-plane vibration of Te-Te bonds, which is mechanically decoupled from the in-plane Fe-Ge bonding network [54]. The weak van der Waals interlayer coupling (~2.95 Å gap) provides structural compliance that buffers the out-of-plane vibration against in-plane strain perturbations. Consequently, despite a large red shift (Δ*ω* ≈ 13.27 cm^−1^) reflecting phonon softening via electron–phonon coupling, the $A_{1g}^{1}$ mode remains observable because the interlayer Te-Te bonding is not directly broken by the interfacial charge transfer. The red shift itself evidences the long-range Coulomb interaction between the charged interface and the Te layers, while the persistence of the mode confirms the structural integrity of the van der Waals stacking. As the dielectric layer increases to 100–300 nm, the gradual re-emergence of the $E_{2g}^{2}$ mode and the diminishing red shift of $A_{1g}^{1}$ (Figure 6b) collectively confirm that the Raman responses are governed by the degree of interfacial charge transfer, which is modulated by dielectric screening. This thickness-dependent phonon behavior establishes an intrinsic link between interfacial charge doping, lattice dynamics, and magnetic properties in the MIS structure.

## 4. Conclusions

Control room temperature magnetism in Fe_3_GeTe_2_ is effectively achieved by adjusting the thickness of the SiO*_x_* dielectric layer. Cross-sectional TEM characterization further reveals a ~5 nm interfacial O-Fe_3_GeTe_2_ oxidation layer that functions as a charge mediation bridge, enhancing the proximity polarization effect particularly for thin dielectric layers where electric field penetration is strong. A thin dielectric layer (50 nm) significantly enhances room temperature ferromagnetism through increased interfacial charge transfer. In contrast, a thick dielectric layer (300 nm) keeps the material nearly intrinsic due to dielectric shielding effects. The study also demonstrates reversible regulation of Fe_3_GeTe_2_ magnetism under UV light irradiation. When exposed to 365 nm UV irradiation, all samples showed varying levels of demagnetization, with the thinnest dielectric layer exhibiting the strongest photoresponse. This light-controlled magnetism is attributed to charge redistribution from photo-generated carriers, suggesting potential for light-controlled spintronic devices. Raman spectroscopy revealed that interfacial coupling affects lattice vibrations. As the dielectric layer thickness increased, the $A_{1g}^{1}$ and $E_{2g}^{2}$ modes exhibited pronounced red shifts, indicating that proximity polarization affects not only the electronic structure but also lattice dynamics through electron–phonon coupling. The correlation model linking dielectric layer thickness, interfacial charge transfer, and magnetic response enhances our understanding of charge–spin coupling in 2D magnetic materials and provides a theoretical basis for designing next-generation silicon-based spintronic devices. Thick dielectric layers are recommended for applications preserving intrinsic material properties, while thin layers are ideal for applications requiring efficient electrically controlled magnetic properties. These findings offer clear guidance for integrating two-dimensional magnetic materials with silicon-based technologies.

## Data Availability

The original contributions presented in this study are included in the article. Further inquiries can be directed to the corresponding author(s).

## Abbreviations

The following abbreviations are used in this manuscript:

2D	Two-dimensional
AFM	Atomic force microscopy
MFM	Magnetic force microscopy
KPFM	Kelvin probe force microscopy
XRD	X-ray diffraction
UV	Ultraviolet
MIS	Metal–insulator–semiconductor
